# Coverage Generosity of Novel Antirheumatic Drugs in Medicare Advantage and Stand‐Alone Part D Plans

**DOI:** 10.1002/art.70132

**Published:** 2026-04-20

**Authors:** Youngmin Kwon, Robert W. Corty, Stacie B. Dusetzina

**Affiliations:** ^1^ Department of Health Policy Vanderbilt University Medical Center Nashville Tennessee; ^2^ Division of Rheumatology and Immunology, Department of Medicine Vanderbilt University Medical Center Nashville Tennessee

## Abstract

**Objective:**

We examine coverage of self‐administered disease‐modifying antirheumatic drugs (DMARDs) in Medicare Part D.

**Methods:**

Using 2022–2026 Part D formulary data, we assessed coverage of the following DMARD classes that treat relapsed rheumatoid arthritis (RA): tumor necrosis factor (TNF) inhibitors, T cell costimulatory modulator, interleukin‐6 (IL‐6) inhibitors, and JAK inhibitors. We measured (1) coverage of individual DMARDs and (2) the generosity of coverage between classes (the share of plans covering at least one drug in each class) and within classes (the share of covered drugs within each class).

**Results:**

We included 26,915 plan‐year observations, representing 217.6 million enrolled beneficiaries from 2022 to 2026. In the five‐year period, coverage was relatively complete (>94%) for etanercept and adalimumab (TNF inhibitors) and upadacitinib (JAK inhibitor). However, coverage was lower for golimumab and certolizumab (TNF inhibitors), abatacept (the sole T cell costimulatory modulator), sarilumab (IL‐6 inhibitor), and baricitinib (JAK inhibitor). Moreover, only 10.4% and 37.6% of stand‐alone and Medicare Advantage (MA) Part D plans, respectively, covered all mechanisms of action, mostly due to low coverage of abatacept. In 2026, only 0.3% and 21.2% of stand‐alone and MA Part D plans, respectively, covered abatacept. On the other hand, coverage increased for IL‐6 inhibitors, likely due to the availability of biosimilar drugs for tocilizumab in 2024.

**Conclusion:**

The growing exclusions of self‐administered DMARDs across several mechanisms of action and heterogeneity in coverage by plan type suggest potential barriers to accessing optimal RA treatments. Further monitoring of formulary adequacy and research into the clinical impacts of formulary exclusions are warranted.

## INTRODUCTION

Clinical management of rheumatoid arthritis (RA) has been greatly improved by the introduction of novel disease‐modifying antirheumatic drugs (DMARDs), including biologic DMARDs and targeted‐synthetic DMARDs. These novel therapies reduce symptom burden and improve health‐related quality of life by delaying joint damage in patients with an inadequate response to conventional synthetic DMARDs that are commonly prescribed as first‐line treatment.^1^, ^2^

Because not all novel DMARDs are equally efficacious or safe for each patient, finding a suitable treatment often involves switching to a DMARD with a different mechanism of action or cycling within the same drug class.^3^ This suggests that having access to a broad range of DMARDs is crucial for optimal RA management. However, the high costs of DMARDs have led health insurers to restrict coverage. For example, in prior studies of coverage in the Medicare Part D program (which administers prescription drug benefits for Medicare beneficiaries), between 69% and 79% of all DMARDs were covered by Part D plans and nearly all drugs were subjected to utilization restrictions, such as prior authorization.^4^, ^5^ Restrictive coverage of DMARDs has fueled concerns that Medicare beneficiaries may face inadequate access to DMARDs,^6^ which may exacerbate the burden of RA among older adults.^7^

Medicare coverage of novel DMARDs is further complicated by the fact that coverage for a specific drug may vary by its route of administration and whether a beneficiary is enrolled in traditional Medicare (TM) versus Medicare Advantage (MA).^8^ Many DMARDs are self‐administered medications, which are covered under Medicare's pharmacy benefit (Part D), whereas other clinician‐administered DMARDs are covered under Medicare's outpatient medical benefit (Part B).^9^ In TM, Part D benefits are separately administered and reimbursed through “stand‐alone” private plans, whereas Part B benefits are reimbursed by the federal Medicare program. In contrast, Part B and Part D benefits are integrated within MA, with reimbursement through a single private MA plan. Given differences in benefit design and preferences for clinician‐administered versus self‐administered medications between MA and stand‐alone plans,^10^, ^11^ coverage of DMARDs may differ by the type of plan in which beneficiaries are enrolled.

Given mounting concerns about inadequate access to novel DMARDs in Medicare, we provide recent insights into self‐administered DMARD coverage in Medicare Part D and compare DMARD coverage across plan types (stand‐alone vs. MA Part D plans). Importantly, our analysis spans years following the Part D benefit redesign under the Inflation Reduction Act (IRA),^12^ which may have generated additional financial pressure to exclude coverage by sharply increasing Part D plans’ responsibility for catastrophic drug costs beyond a newly instituted $2,000 cap on out‐of‐pocket costs. Given the importance of breadth in DMARD coverage, we measured the generosity of coverage by examining how broadly plans cover DMARDs between and within mechanisms of action.

## MATERIALS AND METHODS

### Data and study population

We analyzed Part D plan formulary data from 2022 to 2026,^13^ characterizing coverage of DMARDs in the first quarter of each year, separately by stand‐alone and MA Part D plans. Our study population included plan‐year observations for each stand‐alone or MA Part D plan (identified using unique combinations of the contract and plan identifiers) that were available at any point during this period and contributing to the plan formulary data (data for certain plans are suppressed in the formulary data due to plan requests or data quality issues; see the Supplement for further details). To account for the share of beneficiaries represented by each plan, we also obtained plan enrollment data from the monthly Part D enrollment data. Institutional review board approval was waived because we analyzed publicly available plan‐level data. We analyzed the data from March 2025 to January 2026.

### Eligible DMARDs

We examined coverage of drugs belonging to the following classes of DMARDs that are guideline‐recommended treatments for relapsed RA^2^: tumor necrosis factor (TNF) inhibitors (etanercept, adalimumab, golimumab, and certolizumab pegol), T cell costimulation modulator (abatacept), interleukin‐6 (IL‐6) inhibitors (tocilizumab and sarilumab), and JAK inhibitors (tofacitinib, baricitinib, and upadacitinib). These drugs are available as either subcutaneous or oral formulations, and some also have clinician‐administered formulations. For each plan, we identified coverage for each unique ingredient, dose, and formulation for these products using their RxNorm concept unique identifiers listed in the US Centers for Medicare and Medicaid Services (CMS) Part D formulary reference file^14^, ^15^ (see eTable 1 in the Supplement). Two other DMARDs, infliximab and rituximab, were excluded because they are primarily clinician‐administered medications covered under Part B and are no longer listed in the formulary reference file as of 2020. For drugs with biosimilars (adalimumab and tocilizumab), we considered coverage of either the originator or the biosimilar as coverage for that product (we examined the sensitivity of our results to excluding biosimilar coverage from the analysis).

### Outcomes

We initially determined per‐plan coverage of each individual DMARD. Then, we constructed two measures of coverage generosity. First, we calculated the share of plans covering at least one drug in each class to examine the generosity of coverage across mechanisms of action (ie, between‐class). Second, we calculated the share of drugs covered within each mechanism of action to ascertain the within‐class coverage generosity. For this measure, we divided the number of covered drugs in the class (numerator) by the total number of drugs in the class (denominator).

As another measure of within‐class coverage generosity, we determined the share of plans that cover at least two drugs in each class. For the T cell costimulatory modulator class, which included only a single drug (abatacept), we determined the share of plans covering abatacept. This latter evaluation assesses the adequacy of coverage by “simulating” the Part D formulary requirement that at least two different drugs be covered in each therapeutic class.^16^

### Statistical analysis

We calculated regression‐adjusted differences in outcomes between stand‐alone and MA Part D plans, controlling for year fixed effects, reflecting the average across the six‐year period. All estimates were weighted by plan enrollment in May of each year (up‐weighing plans with a larger number of beneficiaries),^17^ and standard errors were clustered at the plan level to account for within‐plan clustering of outcomes across years. We also compared annual trends in outcomes across plans separately for stand‐alone and MA Part D plans. We analyzed the data using SAS Studio and STATA MP 14.

We conducted several sensitivity analyses. First, we reanalyzed the data for plans available throughout the study period to account for insurer exits from the Part D market, especially following the Part D benefit redesign.^18^ Second, we further excluded Special Needs Plans and regional plans from the sample to focus on representative plans that were available to all Medicare beneficiaries. Third, we replicated the analysis but included two drugs (anakinra and rilonacept^19^, ^20^) belonging to an additional drug class—IL‐1 inhibitor—which is less commonly used and often prescribed as off‐label treatment for RA (we note that canakinumab is another drug in this category but was not included in Part D formularies during the study period). Third, we assessed the use of other formulary restrictions, including utilization management (prior authorization, step therapy, or quantity limits) and placement on a specialty tier, conditional on coverage.

## RESULTS

### Overall pooled differences in DMARDs coverage generosity between stand‐alone and MA Part D plans from 2022 to 2026

We included 26,915 plan‐year observations, representing 217.6 million enrolled beneficiaries from 2022 to 2026 (weighted proportions were 58.2% and 41.8% for stand‐alone and MA Part D plans, respectively). During the five‐year period we examined, we found varying DMARD coverage across regimens and plan types (Table 1). Coverage was nearly universal for etanercept, adalimumab, and upadacitinib (>94% of plans providing coverage). However, coverage was lower for other DMARDs, including golimumab and certolizumab (TNF inhibitors), abatacept (T cell costimulatory modulator), sarilumab (IL‐6 inhibitor), and baricitinib (JAK inhibitor).

**Table 1:** Mean coverage of specific disease‐modifying therapy for relapsed rheumatoid arthritis by stand‐alone and MA Part D plans during 2022–2026*

Coverage, %^a^	Stand‐alone plans, mean (95% CI)	MA plans, mean (95% CI)
TNF inhibitors		
Etanercept	93.9 (92.5–95.4)	96.1 (95.6–96.6)
Adalimumab	100 (100–100)	100 (100–100)
Golimumab	0.1 (0–0.2)	23.8 (21.6–26)
Certolizumab	0.1 (0–0.2)	26.8 (24.6–29)
T cell costimulatory modulator		
Abatacept	19.4 (16.4–22.3)	44.8 (42.1–47.5)
IL‐6 inhibitors		
Tocilizumab	47.9 (44.3–51.5)	58.1 (55.8–60.4)
Sarilumab	14.6 (12.5–16.8)	27.4 (25.3–29.5)
JAK inhibitors		
Tofacitinib	64.9 (61.4–68.4)	73.1 (70.9–75.4)
Baricitinib	0.1 (0–0.2)	9.1 (6.8–11.5)
Upadacitinib	95.1 (93.3–96.9)	99.4 (99.1–99.6)

* CI, confidence interval; IL‐6, interleukin‐6; MA, Medicare Advantage; TNF, tumor necrosis factor.

^a^ The pooled yearly average of plans covering each drug, separately shown for stand‐alone and MA plans, adjusting for secular trends (via year fixed effects) from 2022 to 2026. All estimates were clustered at the plan level to account for within‐plan clustering over the study period and were weighted by plan enrollment in May of each year, giving plans with a larger number of beneficiaries greater weight.

In terms of coverage generosity, 10.4% and 37.6% of stand‐alone and MA Part D plans, respectively, covered all mechanisms of action (Table 2). Among stand‐alone plans, the mean coverage share was 48.5% for TNF inhibitors, 19.4% for T cell costimulatory modulators, 31.3% for IL‐6 inhibitors, and 53.4% for JAK inhibitors. Among MA Part D plans, the mean coverage share was 61.7% for TNF inhibitors, 44.8% for T cell costimulatory modulators, 42.7% for IL‐6 inhibitors, and 60.5% for JAK inhibitors.

**Table 2:** Coverage generosity of disease‐modifying therapy for relapsed rheumatoid arthritis in stand‐alone and MA Part D plans, 2022–2026*

Coverage generosity measures^a^	Stand‐alone plans, mean (95% CI)	MA plans, mean (95% CI)
Covering all mechanism of action, %^b^	10.4 (8.4–12.5)	37.6 (34.9–40.2)
Coverage share of TNF inhibitors (4), %^c^ ^,^ ^d^	48.5 (48.2–48.9)	61.7 (60.5–62.8)
Coverage share of T cell costimulatory modulator (1), %^c^ ^,^ ^e^	19.4 (16.4–22.3)	44.8 (42.1–47.5)
Coverage share of IL‐6 inhibitors (2), %^c^ ^,^ ^f^	31.3 (29.5–33.1)	42.7 (41.2–44.3)
Coverage share of JAK inhibitors (3), %^c^ ^,^ ^g^	53.4 (52.1–54.6)	60.5 (59.3–61.8)

* CI, confidence interval; IL‐6, interleukin‐6; MA, Medicare Advantage; TNF, tumor necrosis factor.

^a^ The table displays the pooled yearly average of the coverage generosity measure for stand‐alone and MA plans, adjusting for secular trends (via year fixed effects) from 2022 to 2026. All estimates were clustered at the plan level to account for within‐plan clustering over the study period and were weighted by plan enrollment in May of each year, giving plans with a larger number of beneficiaries greater weight.

^b^ Proportion of plans covering at least one drug belonging to each mechanism of action.

^c^ Coverage share for each plan was determined by dividing the number of covered drugs in each mechanism of action (numerator) by the total number of drugs belonging to each class (denominator), which is indicated by the number in parentheses.

^d^ TNF inhibitors included etanercept, adalimumab (or its biosimilar), golimumab, or certolizumab pegol.

^e^ Abatacept is the only T cell costimulatory modulator included in the analysis.

^f^ IL‐6 inhibitors included tocilizumab (or its biosimilar drug) and sarilumab.

^g^ JAK inhibitors included tofacitinib, baricitinib, and upadacitinib.

### Trends in DMARDs coverage by plan type

In examining trends in coverage for specific DMARDs, we observed a changing coverage landscape for all DMARDs except etanercept, adalimumab, and upadacitinib in both stand‐alone and MA plans (Figure 1). Among stand‐alone plans, coverage was essentially null for golimumab, certolizumab, and baricitinib throughout 2022 to 2026. The trend in coverage was more volatile for abatacept, sarilumab, and tofacitinib. On the other hand, MA plans had more consistent coverage throughout this period.

**Figure 1:**
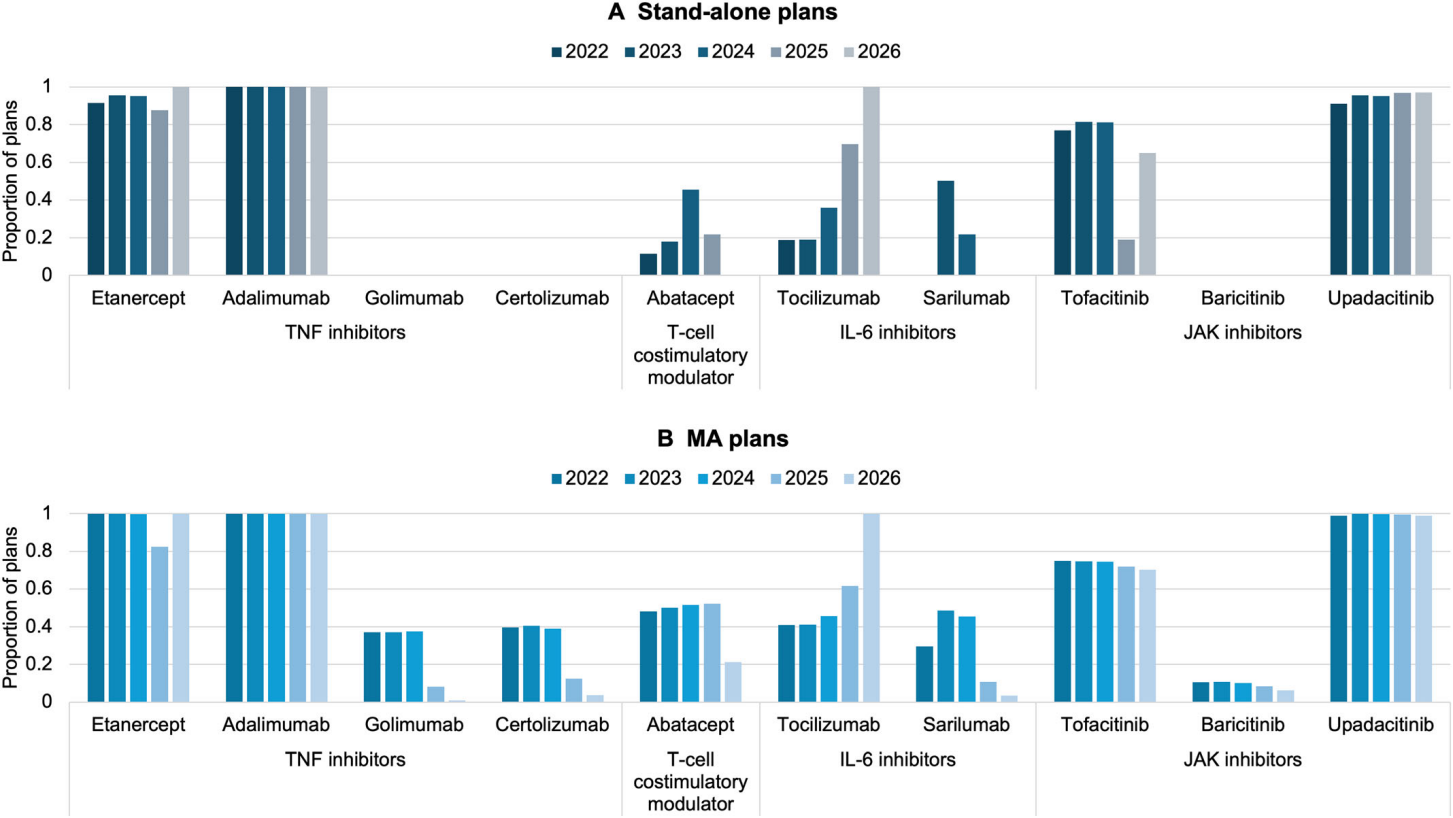
Trends in coverage of specific disease‐modifying therapy for relapsed rheumatoid arthritis in stand‐alone and MA Part D plans. The figure shows yearly trends in coverage for each DMARD under stand‐alone Part D plans (A) and MA Part D plans (B). All estimates were clustered at the plan level to account for within‐plan clustering over the study period and were weighted by plan enrollment in May of each year, giving plans with a larger number of beneficiaries greater weight. DMARD, disease‐modifying antirheumatic drug; IL‐6, interleukin‐6; MA, Medicare Advantage; TNF, tumor necrosis factor.

At the same time, several notable trends emerged in the later years of the study. For instance, there were large drops in coverage of golimumab, certolizumab, abatacept, sarilumab, and baricitinib in 2025 and 2026. For both stand‐alone and MA plans, tocilizumab coverage increased progressively, reaching near‐universal coverage in 2026.

When analyzing the trends in coverage generosity measures, we note that the share of plans covering all mechanisms of action sharply dropped from 11.7% to 0.3% for stand‐alone plans and 42.3% to 21.2% for MA Part D plans, due to many plans excluding abatacept from their formularies from 2025 to 2026 (Figure 2). The trend in coverage share was relatively stable for TNF inhibitors or progressively increasing for IL‐6 inhibitors.

**Figure 2:**
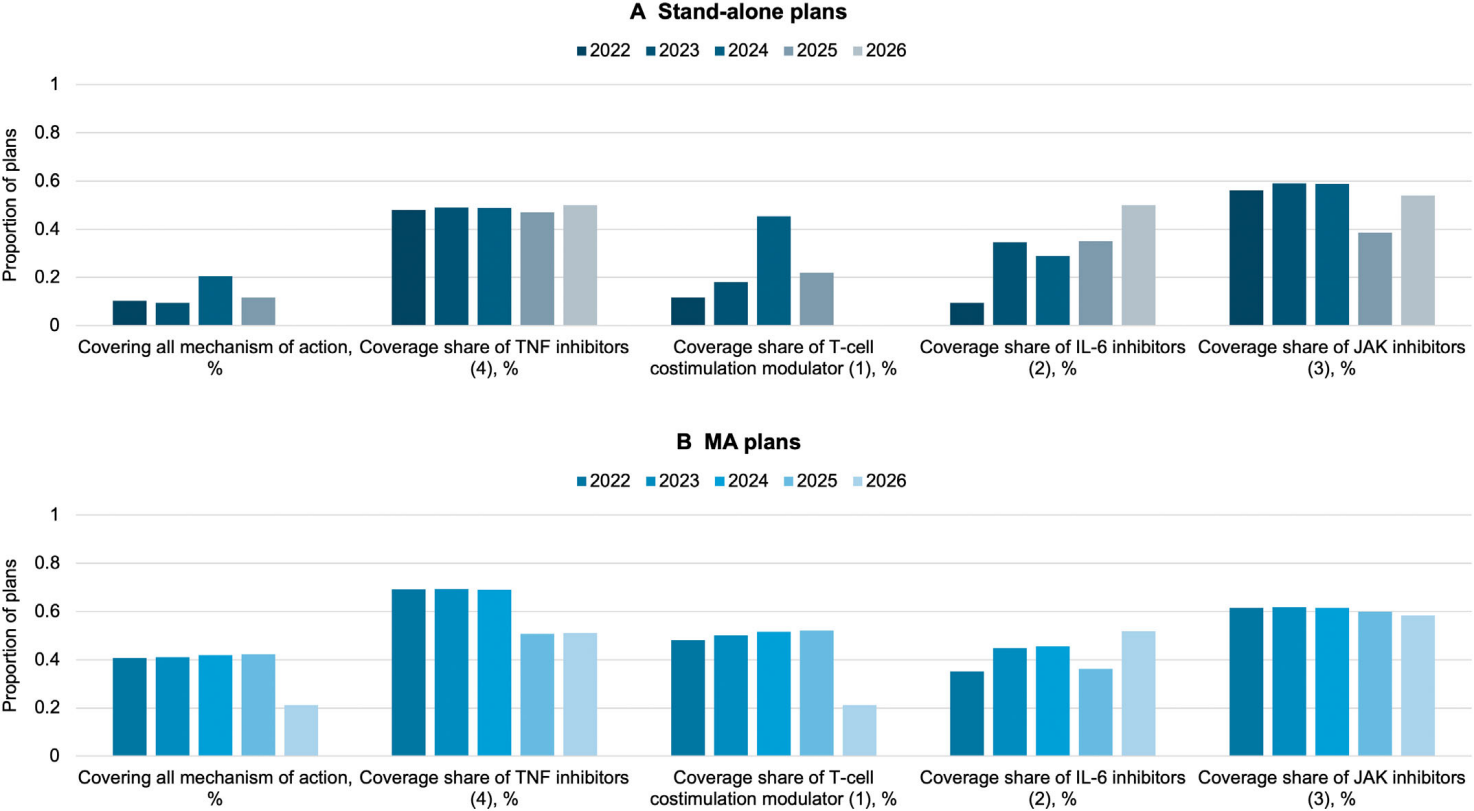
Trends in coverage generosity of disease‐modifying therapy for relapsed rheumatoid arthritis between stand‐alone and MA Part D plans, 2022–2025. The figure presents yearly trends in coverage generosity measures for stand‐alone Part D plans (A) and MA Part D plans (B). To measure the breadth of coverage, we examined the share of plans covering at least one drug in each mechanism of action (TNF inhibitors, T cell costimulatory inhibitors, IL‐6 inhibitors, and JAK inhibitors). We then calculated coverage share for each plan by dividing the number of covered drugs in each mechanism of action (numerator) by the total number of drugs in each class (denominator), as indicated by the number in parentheses. All estimates were clustered at the plan level to account for within‐plan clustering over the study period and were weighted by plan enrollment in May of each year, giving plans with a larger number of beneficiaries greater weight. IL‐6, interleukin‐6; MA, Medicare Advantage; TNF, tumor necrosis factor. Color figure can be viewed in the online issue, which is available at http://onlinelibrary.wiley.com/doi/10.1002/art.70132/abstract.

### Sensitivity analyses

Our results were robust to limiting the sample to plans available throughout the study period (eTable 2) and to representative plans only (eTable 3). Although excluding biosimilars from the analysis did not change our main conclusions, we found that biosimilars accounted for the substantial increase in tocilizumab coverage (eTable 4).

Our finding on between‐class coverage generosity remained consistent when IL‐1 inhibitors were included in our analysis (eTable 5). Because essentially all plans covered at least one IL‐1 inhibitor, inclusion of IL‐1 inhibitors did not impact the estimate of plans covering all mechanisms of action. Notably, there was a sharp increase in coverage of anakinra from 0.3% to 84.1% among stand‐alone plans and 13.3% to 68.6% among MA plans from 2025 to 2026.

Although the use of other formulary restrictions was high across plan types, stand‐alone plans were more likely to apply utilization management and place DMARDs on specialty tiers than MA plans (eTable 6). We also detected some regional variations in coverage; plans operating in the West and Northeast had more generous coverage than plans operating in the Midwest and South (eTable 7).

When applying the minimum threshold of two drugs per class to determine within‐class generosity, we found comparatively high levels of coverage generosity between stand‐alone and MA plans for TNF inhibitors (93.9% and 96.1%, respectively) and JAK inhibitors (60.0% and 72.5%, respectively; eTable 8). For JAK inhibitors, tofacitinib and upadacitinib were the preferred drugs, with virtually no plans covering baricitinib. The share of plans covering both IL‐6 inhibitors was low for both stand‐alone plans (0.1%) and MA plans (9.0%), with most plans exclusively covering tocilizumab rather than sarilumab.

## DISCUSSION

In this national study of Medicare Part D plan formularies, we observed general trends of reduced coverage of novel, self‐administered DMARDs treating relapsed RA from 2022 to 2026. In 2026, there was a noticeable drop in plans covering abatacept, the sole T cell costimulatory inhibitor, which considerably reduced the share of plans covering all mechanisms of action (with few to no stand‐alone plans covering abatacept in 2026). Moreover, we found low shares of plans covering golimumab and certolizumab (TNF inhibitors), sarilumab (IL‐6 inhibitor), and baricitinib (JAK inhibitor). In contrast, coverage of tocilizumab has substantially increased in 2026, likely driven by the launch of its biosimilar in 2024.^21^ In general, the coverage of DMARDs was more generous among MA plans as opposed to stand‐alone plans, both across and within classes.

This declining coverage of DMARDs, as well as the differential coverage across plan types, has access implications for beneficiaries who need such treatments. In clinical practice, switching to alternative DMARDS between and within drug classes to address treatment failure and nonresponse or to avoid contraindicated medications is increasingly common.^22^, ^23^ As such, formularies that restrict access to a wide range of DMARDs can limit physicians’ and patients’ autonomy in selecting the optimal care regimen. Moreover, beneficiaries who must switch from self‐administered to clinician‐administered DMARDs (due to Part D coverage exclusions) may face the time burden of regular infusions at physician practices and high out‐of‐pocket costs, especially if they are in TM without supplemental coverage.^24^ Broadly, limited coverage of self‐administered DMARDs may circumvent the goal of the Part D benefit redesign under the IRA to provide financial relief for Medicare beneficiaries filling high‐cost drugs. Because the newly enacted $2,000 cap on out‐of‐pocket costs only applies to drugs covered by Part D formularies,^25^ beneficiaries treated with DMARDs not covered by their plans may still experience access and affordability problems.

Given these observed changes in coverage, it is imperative that beneficiaries compare Part D plan options during open enrollment to ensure their plan includes the preferred DMARDs.^26^ However, because most beneficiaries do not shop for plans after their initial enrollment decisions^12^, ^27^, ^28^ and the number of available plans offering comprehensive coverage has decreased, many beneficiaries may remain in plans that may not provide optimal coverage. The search for optimal coverage is further complicated by the fact that patients may not know which DMARD(s) will be indicated for their use in the year(s) ahead. Given these facts, future studies should continue to examine how the Part D formulary evolves over time and illuminate any potential barriers to access and their impacts on patient outcomes.

Our study—focusing specifically on gaps in RA drug coverage—has larger implications for the adequacy of Part D formularies. The finding that plans issued narrowed coverage for RA drugs comports with the general trend of Part D plans covering fewer drugs over time (among all drugs eligible for Part D coverage), with the share of covered drugs decreasing from 73% in 2010 to 54% in 2024.^29^ In providing additional evidence of this fact, we also highlight the differential trends in coverage generosity between stand‐alone and MA plans. It is possible that these trends reflect diverging plan preferences for self‐administered versus clinician‐administered drugs, especially given clinical exchangeability between these formulations.^10^, ^30^ For instance, stand‐alone plans may prefer less generous coverage of self‐administered DMARDs in Part D to shift beneficiaries toward clinician‐administered drugs because they are only financially responsible for managing the pharmacy benefit. On the other hand, MA plans manage spending for both pharmacy and medical benefits and may prefer self‐administered medications to reduce the administrative costs associated with infusions. In addition to these financial factors, MA plans may be weighing the safety and efficacy of clinician‐administered versus self‐administered medications, as well as preferences for different formulations, within their respective patient populations and among providers participating in their network.^10^, ^31^ With an increasing number of therapeutically equivalent medications that vary by routes of administration (eg, Ig treatments and interferon therapies for multiple sclerosis),^10^, ^32^ we may expect further heterogeneity in formulary coverage between stand‐alone and MA Part D plans for these drugs. This underscores the need for additional research examining potential variations in coverage across plan types and the causal mechanisms underlying these variations.

Addressing the trend toward restrictive formularies may require regulators to revisit how they ascertain formulary adequacy. Statutorily, a key requirement of Part D formularies is the mandate to cover at least two drugs within therapeutic categories and classes. This provides some floor for coverage and allows CMS to identify formularies with egregious coverage gaps.^33^, ^34^ At the same time, plans have ample latitude in defining therapeutic categories and classes^35^; outside the six protected drug classes in which plans are required to cover “all or substantially all” drugs,^36^ plans retain considerable freedom in determining the scope of coverage. To illustrate this issue in our context, we note that all included drugs in our study are listed under a very broad therapeutic category (“immunological agents” or “immunosuppressant”) in the US Pharmacopeia, one of the drug classification systems used in formulary reviews.^16^ Because this category encompasses many biologic medications, not all of which treat the same condition (like RA), the requirement to cover at least two drugs in this category does not necessarily translate into comprehensive coverage of RA drugs. Hypothetically, if drugs were categorized by their exact mechanisms of action (eg, T cell costimulatory modulator) for a particular condition, such as RA, as we have done in our simulation analysis, then formularies may face stricter minimum coverage requirements. Thus, the definition for therapeutic categories and classes may need to be reviewed to ensure adequate coverage for distinct conditions.

However, there are important trade‐offs to mandating more comprehensive DMARD coverage. Imposing a requirement for broader coverage may preclude plans from using the prospect of formulary exclusion as leverage when negotiating price concessions from manufacturers. In a prior study, the average amount of rebates (ie, price discounts) for drugs belonging to the protected classes in Part D is lower than that for drugs in non‐protected classes,^36^ suggesting that more generous coverage of high‐cost specialty medications such as DMARDs may come at the expense of increased drug spending by Medicare and its beneficiaries. This may also run counter to the desire of Part D plans to more tightly control formularies,^37^, ^38^ especially in the post‐IRA period when plans are responsible for a greater share of catastrophic drug spending,^12^ and may induce further volatility in the Part D market.^18^ At the same time, it is necessary to account for the nuances of specific clinical contexts when ascertaining and weighting such trade‐offs. In RA, exclusion of a single product could be clinically consequential because patients often receive multiple lines of therapy,^39^ providing a stronger clinical rationale for ensuring broader coverage relative to other therapeutic areas featuring more substitutable treatments. Taken together, a more targeted, granular assessment of formulary adequacy may be needed to inform efforts to optimize coverage across drug classes.

We acknowledge several limitations. First, the descriptive nature of the study precludes any causal explanations for why stand‐alone and MA Part D plans may cover self‐administered DMARDs differently. Instead, the goal of this research was to generate hypotheses behind variations in coverage. Second, whether the observed coverage differences translate into actual differences in utilization and patient outcomes remains unknown but is a fruitful avenue for future research. Third, we did not estimate the costs of DMARDs across plan options and were unable to compare costs by treatment because they depend on specific plan benefit structure and the decision to use self‐administered versus clinician‐administered DMARDs. Fourth, because our results may not generalize to other therapeutic contexts, future studies investigating coverage disparities for other high‐cost medications are needed to provide a more comprehensive understanding of formulary adequacy in Part D.

In this national study of Medicare Part D formularies, we found reductions in coverage of novel self‐administered DMARDs in several mechanisms of action in recent periods and heterogeneity in coverage generosity between stand‐alone and MA Part D plans, which may impact patient access to and affordability of RA treatment. Our findings underscore the importance of monitoring the adequacy of Part D formularies more closely, especially considering recent policy changes, such as the IRA, that have significantly impacted the Part D program.

## AUTHOR CONTRIBUTIONS

All authors contributed to at least one of the following manuscript preparation roles: conceptualization AND/OR methodology, software, investigation, formal analysis, data curation, visualization, and validation AND drafting or reviewing/editing the final draft. As corresponding author, Dr Kwon confirms that all authors have provided the final approval of the version to be published and takes responsibility for the affirmations regarding article submission (eg, not under consideration by another journal), the integrity of the data presented, and the statements regarding compliance with institutional review board/Declaration of Helsinki requirements.

## Supporting information


**Disclosure form**.

**Table S1:** List of included DMARDs and RXCUI codes used for identification
**Table S2:** Sensitivity to limiting the sample to plans operating throughout the study period
**Table S3:** Sensitivity of main estimates to excluding non‐representative plans
**Table S4:** Sensitivity of main estimates to excluding biosimilars
**Table S5:** Sensitivity of main estimates to including IL‐1 inhibitors
**Table S6:** Other restrictions on covered drugs by plan type
**Table S7:** Stratified analysis by Census region
**Table S8:** Using “at least 2 drugs” threshold to evaluate within‐class generosity
