# Supplementary material for: Coverage Generosity of Novel Antirheumatic Drugs in Medicare Advantage and Stand‐Alone Part D Plans
Source: Arthritis Rheumatol. 2026 Apr 20;78(10):2171–8. doi: 10.1002/art.70132 (PMC13619088; doi:10.1002/art.70132)
Supplement: Supplementary file 2 [file ART-78-2171-s002.pdf]

Online Supplemental Appendix

Kwon Y, Corty RW, Dusetzina SB. Coverage generosity of novel anti-rheumatic drugs in Medicare Advantage and stand-alone Part D plans.

eTable 1 List of included DMARDs and RXCUI codes used for identification 4
eTable 2 Sensitivity to limiting the sample to plans operating throughout the study period........12
eTable 3 Sensitivity of main estimates to excluding non-representative plans 13
eTable 4 Sensitivity of main estimates to excluding biosimilars...14
eTable 5 Sensitivity of main estimates to including IL-1 inhibitors..15
eTable 6 Other restrictions on covered drugs by plan type 16
eTable 7 Stratified analysis by Census region...17
eTable 8 Using "at least 2 drugs" threshold to evaluate within-class generosity 18

eMethods. Identification of DMARDs coverage and other utilization restrictions

To ascertain coverage of self-administered DMARDs among Part D plans, we analyzed the public use Part D formulary files for years 2022 to 2026, released by Centers for Medicare and Medicaid Services (CMS).[1] The formulary files contain plan information (plan name and identifiers and type) and basic formulary details for each plan, including coverage, use of utilization management, and drug tiers. We identified a unique plan as a combination of CONTRACT_ID, PLAN_ID, and SEGMENT_ID (which identifies the unique geographic market). We excluded a small number of plans whose data are suppressed in the public use files due to plan request and data quality issues (these plans constitute <1% of all Part D plans in each year).

In the formulary files, we identify coverage of each included DMARD using RxNorm Concept Unique Identifiers (RXCUIs), which encompass the unique ingredient, dose, and formulation for products.[16] We obtained the list of RXCUI codes for included DMARDs from the CMS Formulary Reference File, containing a list of drugs that may be included in Part D formularies.[16] The full list of RXCUIs employed in the analysis is shown in (**eTable 1**). We considered the plan to cover each DMARD if the plan formulary listed at least one of the RXCUI associated with the drug. We included RXCUIs for both originator and biosimilar products (when available), considering coverage of either product as having coverage for a given DMARD.

Our outcome measures examined the *generosity* of coverage between and within each following DMARD class:

- **Tumor Necrosis Factor (TNF) inhibitors**: etanercept, adalimumab (and its biosimilars), golimumab, and certolizumab pegol

- **T-cell co-stimulatory modulator**: abatacept (a sole drug in the class)
- **Interleukin-6 inhibitors**: tocilizumab and sarilumab
- **Janus Kinases (JAK) inhibitors**: tofacitinib, baricitinib, and upadacitinib
- **Interleukin-1 (IL-1) inhibitors**: anakinra and rilonacept (only evaluated in a sensitivity analysis because they are less commonly used and often prescribed as off-label treatment[21,22]).

We also examined other dimensions of coverage restrictions—use of any utilization management (i.e., prior authorization, step therapy, or quantity limit) and the placement of drugs in the special tier (i.e., tier 5 or 6)—conditional on coverage. They were determined based on the following variables in the formulary files: PRIOR_AUTHORIZATION_YN, QUANTITY_LIMIT_YN, STEP_THERAPY_YN, and TIER_LEVEL_VALUE.

eTable 1 List of included DMARDs and RXCUI codes used for identification

Drug	Drug ingredient, dose, strength	RXCUI	Year
Abatacept	1 Ml Abatacept 125 Mg/Ml Prefilled Syringe [Orencia]	1145932	2022
Abatacept	1 Ml Abatacept 125 Mg/Ml Auto-Injector [Orencia]	1799230	2022
Abatacept	0.4 Ml Abatacept 125 Mg/Ml Prefilled Syringe [Orencia]	1925255	2022
Abatacept	0.7 Ml Abatacept 125 Mg/Ml Prefilled Syringe [Orencia]	1925257	2022
Abatacept	1 Ml Abatacept 125 Mg/Ml Prefilled Syringe [Orencia]	1145932	2023
Abatacept	1 Ml Abatacept 125 Mg/Ml Auto-Injector [Orencia]	1799230	2023
Abatacept	0.4 Ml Abatacept 125 Mg/Ml Prefilled Syringe [Orencia]	1925255	2023
Abatacept	0.7 Ml Abatacept 125 Mg/Ml Prefilled Syringe [Orencia]	1925257	2023
Abatacept	1 Ml Abatacept 125 Mg/Ml Prefilled Syringe [Orencia]	1145932	2024
Abatacept	1 Ml Abatacept 125 Mg/Ml Auto-Injector [Orencia]	1799230	2024
Abatacept	0.4 Ml Abatacept 125 Mg/Ml Prefilled Syringe [Orencia]	1925255	2024
Abatacept	0.7 Ml Abatacept 125 Mg/Ml Prefilled Syringe [Orencia]	1925257	2024
Abatacept	1 Ml Abatacept 125 Mg/Ml Prefilled Syringe [Orencia]	1145932	2025
Abatacept	1 Ml Abatacept 125 Mg/Ml Auto-Injector [Orencia]	1799230	2025
Abatacept	0.4 Ml Abatacept 125 Mg/Ml Prefilled Syringe [Orencia]	1925255	2025
Abatacept	0.7 Ml Abatacept 125 Mg/Ml Prefilled Syringe [Orencia]	1925257	2025
Adalimumab	0.8 Ml Adalimumab 50 Mg/Ml Prefilled Syringe [Humira]	727705	2022
Adalimumab	{6 (0.8 Ml Adalimumab 50 Mg/Ml Auto-Injector [Humira]) } Pack [Humira Pen - Cr	825170	2022
Adalimumab	0.8 Ml Adalimumab 50 Mg/Ml Auto-Injector [Humira]	1655728	2022
Adalimumab	0.4 Ml Adalimumab 100 Mg/Ml Prefilled Syringe [Humira]	1726846	2022
Adalimumab	{4 (0.8 Ml Adalimumab 50 Mg/Ml Auto-Injector [Humira]) } Pack [Humira Pen - Ps	1801278	2022
Adalimumab	{1 (0.4 Ml Adalimumab 100 Mg/Ml Prefilled Syringe [Humira]) / 1 (0.8 Ml Adalim	1855527	2022
Adalimumab	0.4 Ml Adalimumab 100 Mg/Ml Auto-Injector [Humira]	1872980	2022
Adalimumab	{3 (0.8 Ml Adalimumab 100 Mg/Ml Prefilled Syringe [Humira]) } Pack [Humira Pre	1873087	2022
Adalimumab	0.1 Ml Adalimumab 100 Mg/Ml Prefilled Syringe [Humira]	1921010	2022
Adalimumab	0.2 Ml Adalimumab 100 Mg/Ml Prefilled Syringe [Humira]	1921017	2022
Adalimumab	0.8 Ml Adalimumab 100 Mg/Ml Auto-Injector [Humira]	1921240	2022
Adalimumab	{3 (0.8 Ml Adalimumab 100 Mg/Ml Auto-Injector [Humira]) } Pack [Humira Pen 80	1921245	2022
Adalimumab	{2 (0.4 Ml Adalimumab 100 Mg/Ml Auto-Injector [Humira]) / 1 (0.8 Ml Adalimumab	1921468	2022
Adalimumab	{4 (0.8 Ml Adalimumab 100 Mg/Ml Auto-Injector [Humira]) } Pack [Humira Pen 80	2531134	2022
Adalimumab	0.8 Ml Adalimumab 50 Mg/Ml Prefilled Syringe [Humira]	727705	2023
Adalimumab	{6 (0.8 Ml Adalimumab 50 Mg/Ml Auto-Injector [Humira]) } Pack [Humira Pen - Cr	825170	2023
Adalimumab	0.8 Ml Adalimumab 50 Mg/Ml Auto-Injector [Humira]	1655728	2023
Adalimumab	0.4 Ml Adalimumab 100 Mg/Ml Prefilled Syringe [Humira]	1726846	2023
Adalimumab	{4 (0.8 Ml Adalimumab 50 Mg/Ml Auto-Injector [Humira]) } Pack [Humira Pen - Ps	1801278	2023
Adalimumab	{1 (0.4 Ml Adalimumab 100 Mg/Ml Prefilled Syringe [Humira]) / 1 (0.8 Ml Adalim	1855527	2023
Adalimumab	0.4 Ml Adalimumab 100 Mg/Ml Auto-Injector [Humira]	1872980	2023

Adalimumab	{3 (0.8 Ml Adalimumab 100 Mg/Ml Prefilled Syringe [Humira]) } Pack [Humira Pre	1873087	2023
Adalimumab	0.1 Ml Adalimumab 100 Mg/Ml Prefilled Syringe [Humira]	1921010	2023
Adalimumab	0.2 Ml Adalimumab 100 Mg/Ml Prefilled Syringe [Humira]	1921017	2023
Adalimumab	0.8 Ml Adalimumab 100 Mg/Ml Auto-Injector [Humira]	1921240	2023
Adalimumab	{3 (0.8 Ml Adalimumab 100 Mg/Ml Auto-Injector [Humira]) } Pack [Humira Pen 80	1921245	2023
Adalimumab	{2 (0.4 Ml Adalimumab 100 Mg/Ml Auto-Injector [Humira]) / 1 (0.8 Ml Adalimumab	1921468	2023
Adalimumab	{4 (0.8 Ml Adalimumab 100 Mg/Ml Auto-Injector [Humira]) } Pack [Humira Pen 80	2531134	2023
Adalimumab	0.8 Ml Adalimumab 50 Mg/Ml Prefilled Syringe [Humira]	727705	2024
Adalimumab	{6 (0.8 Ml Adalimumab 50 Mg/Ml Auto-Injector [Humira]) } Pack [Humira Pen - Cr	825170	2024
Adalimumab	0.8 Ml Adalimumab 50 Mg/Ml Auto-Injector [Humira]	1655728	2024
Adalimumab	0.4 Ml Adalimumab 100 Mg/Ml Prefilled Syringe [Humira]	1726846	2024
Adalimumab	{4 (0.8 Ml Adalimumab 50 Mg/Ml Auto-Injector [Humira]) } Pack [Humira Pen - Ps	1801278	2024
Adalimumab	{1 (0.4 Ml Adalimumab 100 Mg/Ml Prefilled Syringe [Humira]) / 1 (0.8 Ml Adalim	1855527	2024
Adalimumab	0.4 Ml Adalimumab 100 Mg/Ml Auto-Injector [Humira]	1872980	2024
Adalimumab	{3 (0.8 Ml Adalimumab 100 Mg/Ml Prefilled Syringe [Humira]) } Pack [Humira Pre	1873087	2024
Adalimumab	0.1 Ml Adalimumab 100 Mg/Ml Prefilled Syringe [Humira]	1921010	2024
Adalimumab	0.2 Ml Adalimumab 100 Mg/Ml Prefilled Syringe [Humira]	1921017	2024
Adalimumab	0.8 Ml Adalimumab 100 Mg/Ml Auto-Injector [Humira]	1921240	2024
Adalimumab	{3 (0.8 Ml Adalimumab 100 Mg/Ml Auto-Injector [Humira]) } Pack [Humira Pen 80	1921245	2024
Adalimumab	{2 (0.4 Ml Adalimumab 100 Mg/Ml Auto-Injector [Humira]) / 1 (0.8 Ml Adalimumab	1921468	2024
Adalimumab	{4 (0.8 Ml Adalimumab 100 Mg/Ml Auto-Injector [Humira]) } Pack [Humira Pen 80	2531134	2024
Adalimumab	0.4 Ml Adalimumab-Atto 50 Mg/Ml Prefilled Syringe [Amjevita]	2627717	2024
Adalimumab	0.8 Ml Adalimumab-Atto 50 Mg/Ml Auto-Injector [Amjevita]	2627722	2024
Adalimumab	0.8 Ml Adalimumab-Atto 50 Mg/Ml Prefilled Syringe [Amjevita]	2627725	2024
Adalimumab	0.2 Ml Adalimumab-Atto 50 Mg/Ml Prefilled Syringe [Amjevita]	2634479	2024
Adalimumab	0.4 Ml Adalimumab-Aaty 100 Mg/Ml Auto-Injector [Yuflyma]	2639657	2024
Adalimumab	0.4 Ml Adalimumab-Aaty 100 Mg/Ml Prefilled Syringe [Yuflyma]	2639662	2024
Adalimumab	0.8 Ml Adalimumab-Bwwd 50 Mg/Ml Auto-Injector [Hadlima]	2640300	2024
Adalimumab	0.8 Ml Adalimumab-Bwwd 50 Mg/Ml Prefilled Syringe [Hadlima]	2640305	2024
Adalimumab	0.8 Ml Adalimumab-Aqvh 50 Mg/Ml Auto-Injector [Yusimry]	2640400	2024
Adalimumab	0.8 Ml Adalimumab-Fkjp 50 Mg/Ml Auto-Injector	2640404	2024
Adalimumab	0.8 Ml Adalimumab-Fkjp 50 Mg/Ml Auto-Injector [Hulio]	2640410	2024
Adalimumab	0.4 Ml Adalimumab-Fkjp 50 Mg/Ml Prefilled Syringe	2640412	2024
Adalimumab	0.4 Ml Adalimumab-Fkjp 50 Mg/Ml Prefilled Syringe [Hulio]	2640415	2024
Adalimumab	0.8 Ml Adalimumab-Fkjp 50 Mg/Ml Prefilled Syringe	2640417	2024
Adalimumab	0.8 Ml Adalimumab-Fkjp 50 Mg/Ml Prefilled Syringe [Hulio]	2640418	2024
Adalimumab	0.4 Ml Adalimumab-Bwwd 100 Mg/Ml Prefilled Syringe [Hadlima]	2640428	2024
Adalimumab	0.4 Ml Adalimumab-Bwwd 100 Mg/Ml Auto-Injector [Hadlima]	2640432	2024
Adalimumab	0.8 Ml Adalimumab-Adbm 50 Mg/Ml Prefilled Syringe [Cyltezo]	2640889	2024
Adalimumab	0.2 Ml Adalimumab-Adbm 50 Mg/Ml Prefilled Syringe [Cyltezo]	2640892	2024

Adalimumab	0.4 Ml Adalimumab-Adbm 50 Mg/Ml Prefilled Syringe [Cyltezo]	2640894	2024
Adalimumab	0.8 Ml Adalimumab-Adbm 50 Mg/Ml Auto-Injector [Cyltezo]	2640898	2024
Adalimumab	{4 (0.8 Ml Adalimumab-Adbm 50 Mg/Ml Auto-Injector [Cyltezo]) } Pack [Cyltezo P	2640901	2024
Adalimumab	{6 (0.8 Ml Adalimumab-Adbm 50 Mg/Ml Auto-Injector [Cyltezo]) } Pack [Cyltezo P	2640903	2024
Adalimumab	0.1 Ml Adalimumab-Adaz 100 Mg/Ml Prefilled Syringe [Hyrimoz]	2641650	2024
Adalimumab	0.2 Ml Adalimumab-Adaz 100 Mg/Ml Prefilled Syringe [Hyrimoz]	2641654	2024
Adalimumab	0.4 Ml Adalimumab-Adaz 100 Mg/Ml Auto-Injector	2641655	2024
Adalimumab	0.4 Ml Adalimumab-Adaz 100 Mg/Ml Auto-Injector [Hyrimoz]	2641658	2024
Adalimumab	0.4 Ml Adalimumab-Adaz 100 Mg/Ml Prefilled Syringe	2641660	2024
Adalimumab	0.4 Ml Adalimumab-Adaz 100 Mg/Ml Prefilled Syringe [Hyrimoz]	2641661	2024
Adalimumab	0.8 Ml Adalimumab-Adaz 100 Mg/Ml Auto-Injector [Hyrimoz]	2641663	2024
Adalimumab	{3 (0.8 Ml Adalimumab-Adaz 100 Mg/Ml Auto-Injector [Hyrimoz]) } Pack [Hyrimoz	2641665	2024
Adalimumab	{3 (0.8 Ml Adalimumab-Adaz 100 Mg/Ml Prefilled Syringe [Hyrimoz]) } Pack [Hyri	2641669	2024
Adalimumab	{2 (0.4 Ml Adalimumab-Adaz 100 Mg/Ml Auto-Injector [Hyrimoz]) / 1 (0.8 Ml Adal	2641671	2024
Adalimumab	{1 (0.4 Ml Adalimumab-Adaz 100 Mg/Ml Prefilled Syringe [Hyrimoz]) / 1 (0.8 Ml	2641673	2024
Adalimumab	0.8 Ml Adalimumab 50 Mg/Ml Prefilled Syringe [Humira]	727705	2025
Adalimumab	0.8 Ml Adalimumab 50 Mg/Ml Auto-Injector [Humira]	1655728	2025
Adalimumab	0.4 Ml Adalimumab 100 Mg/Ml Prefilled Syringe [Humira]	1726846	2025
Adalimumab	0.4 Ml Adalimumab 100 Mg/Ml Auto-Injector [Humira]	1872980	2025
Adalimumab	0.1 Ml Adalimumab 100 Mg/Ml Prefilled Syringe [Humira]	1921010	2025
Adalimumab	0.2 Ml Adalimumab 100 Mg/Ml Prefilled Syringe [Humira]	1921017	2025
Adalimumab	0.8 Ml Adalimumab 100 Mg/Ml Auto-Injector [Humira]	1921240	2025
Adalimumab	{3 (0.8 Ml Adalimumab 100 Mg/Ml Auto-Injector [Humira]) } Pack [Humira Pen 80	1921245	2025
Adalimumab	{2 (0.4 Ml Adalimumab 100 Mg/Ml Auto-Injector [Humira]) / 1 (0.8 Ml Adalimumab	1921468	2025
Adalimumab	{4 (0.8 Ml Adalimumab 100 Mg/Ml Auto-Injector [Humira]) } Pack [Humira Pen 80	2531134	2025
Adalimumab	0.4 Ml Adalimumab-Atto 50 Mg/Ml Prefilled Syringe [Amjevita]	2627717	2025
Adalimumab	0.8 Ml Adalimumab-Atto 50 Mg/Ml Auto-Injector [Amjevita]	2627722	2025
Adalimumab	0.8 Ml Adalimumab-Atto 50 Mg/Ml Prefilled Syringe [Amjevita]	2627725	2025
Adalimumab	0.2 Ml Adalimumab-Atto 50 Mg/Ml Prefilled Syringe [Amjevita]	2634479	2025
Adalimumab	0.4 Ml Adalimumab-Aaty 100 Mg/Ml Auto-Injector	2639651	2025
Adalimumab	0.4 Ml Adalimumab-Aaty 100 Mg/Ml Auto-Injector [Yuflyma]	2639657	2025
Adalimumab	0.4 Ml Adalimumab-Aaty 100 Mg/Ml Prefilled Syringe	2639659	2025
Adalimumab	0.4 Ml Adalimumab-Aaty 100 Mg/Ml Prefilled Syringe [Yuflyma]	2639662	2025
Adalimumab	0.8 Ml Adalimumab-Bwwd 50 Mg/Ml Auto-Injector [Hadlima]	2640300	2025
Adalimumab	0.8 Ml Adalimumab-Bwwd 50 Mg/Ml Prefilled Syringe [Hadlima]	2640305	2025
Adalimumab	0.8 Ml Adalimumab-Aqvh 50 Mg/Ml Auto-Injector [Yusimry]	2640400	2025
Adalimumab	0.8 Ml Adalimumab-Fkjp 50 Mg/Ml Auto-Injector	2640404	2025
Adalimumab	0.8 Ml Adalimumab-Fkjp 50 Mg/Ml Auto-Injector [Hulio]	2640410	2025
Adalimumab	0.4 Ml Adalimumab-Fkjp 50 Mg/Ml Prefilled Syringe	2640412	2025
Adalimumab	0.4 Ml Adalimumab-Fkjp 50 Mg/Ml Prefilled Syringe [Hulio]	2640415	2025

Adalimumab	0.8 Ml Adalimumab-Fkjp 50 Mg/Ml Prefilled Syringe	2640417	2025
Adalimumab	0.8 Ml Adalimumab-Fkjp 50 Mg/Ml Prefilled Syringe [Hulio]	2640418	2025
Adalimumab	0.4 Ml Adalimumab-Bwwd 100 Mg/Ml Prefilled Syringe [Hadlima]	2640428	2025
Adalimumab	0.4 Ml Adalimumab-Bwwd 100 Mg/Ml Auto-Injector [Hadlima]	2640432	2025
Adalimumab	0.8 Ml Adalimumab-Aacf 50 Mg/Ml Prefilled Syringe [Idacio]	2640857	2025
Adalimumab	0.8 Ml Adalimumab-Aacf 50 Mg/Ml Auto-Injector	2640859	2025
Adalimumab	0.8 Ml Adalimumab-Aacf 50 Mg/Ml Auto-Injector [Idacio]	2640862	2025
Adalimumab	{4 (0.8 Ml Adalimumab-Aacf 50 Mg/Ml Auto-Injector [Idacio]) } Pack [Idacio Pen	2640865	2025
Adalimumab	{6 (0.8 Ml Adalimumab-Aacf 50 Mg/Ml Auto-Injector [Idacio]) } Pack [Idacio Pen	2640870	2025
Adalimumab	0.8 Ml Adalimumab-Adbm 50 Mg/Ml Prefilled Syringe	2640883	2025
Adalimumab	0.8 Ml Adalimumab-Adbm 50 Mg/Ml Prefilled Syringe [Cyltezo]	2640889	2025
Adalimumab	0.2 Ml Adalimumab-Adbm 50 Mg/Ml Prefilled Syringe	2640891	2025
Adalimumab	0.2 Ml Adalimumab-Adbm 50 Mg/Ml Prefilled Syringe [Cyltezo]	2640892	2025
Adalimumab	0.4 Ml Adalimumab-Adbm 50 Mg/Ml Prefilled Syringe	2640893	2025
Adalimumab	0.4 Ml Adalimumab-Adbm 50 Mg/Ml Prefilled Syringe [Cyltezo]	2640894	2025
Adalimumab	0.8 Ml Adalimumab-Adbm 50 Mg/Ml Auto-Injector	2640895	2025
Adalimumab	0.8 Ml Adalimumab-Adbm 50 Mg/Ml Auto-Injector [Cyltezo]	2640898	2025
Adalimumab	{4 (0.8 Ml Adalimumab-Adbm 50 Mg/Ml Auto-Injector) } Pack	2640900	2025
Adalimumab	{4 (0.8 Ml Adalimumab-Adbm 50 Mg/Ml Auto-Injector [Cyltezo]) } Pack [Cyltezo P	2640901	2025
Adalimumab	{6 (0.8 Ml Adalimumab-Adbm 50 Mg/Ml Auto-Injector) } Pack	2640902	2025
Adalimumab	{6 (0.8 Ml Adalimumab-Adbm 50 Mg/Ml Auto-Injector [Cyltezo]) } Pack [Cyltezo P	2640903	2025
Adalimumab	0.1 Ml Adalimumab-Adaz 100 Mg/Ml Prefilled Syringe [Hyrimoz]	2641650	2025
Adalimumab	0.2 Ml Adalimumab-Adaz 100 Mg/Ml Prefilled Syringe [Hyrimoz]	2641654	2025
Adalimumab	0.4 Ml Adalimumab-Adaz 100 Mg/Ml Auto-Injector	2641655	2025
Adalimumab	0.4 Ml Adalimumab-Adaz 100 Mg/Ml Auto-Injector [Hyrimoz]	2641658	2025
Adalimumab	0.4 Ml Adalimumab-Adaz 100 Mg/Ml Prefilled Syringe	2641660	2025
Adalimumab	0.4 Ml Adalimumab-Adaz 100 Mg/Ml Prefilled Syringe [Hyrimoz]	2641661	2025
Adalimumab	0.8 Ml Adalimumab-Adaz 100 Mg/Ml Auto-Injector [Hyrimoz]	2641663	2025
Adalimumab	{3 (0.8 Ml Adalimumab-Adaz 100 Mg/Ml Auto-Injector [Hyrimoz]) } Pack [Hyrimoz	2641665	2025
Adalimumab	{3 (0.8 Ml Adalimumab-Adaz 100 Mg/Ml Prefilled Syringe [Hyrimoz]) } Pack [Hyri	2641669	2025
Adalimumab	{2 (0.4 Ml Adalimumab-Adaz 100 Mg/Ml Auto-Injector [Hyrimoz]) / 1 (0.8 Ml Adal	2641671	2025
Adalimumab	{1 (0.4 Ml Adalimumab-Adaz 100 Mg/Ml Prefilled Syringe [Hyrimoz]) / 1 (0.8 Ml	2641673	2025
Adalimumab	0.2 Ml Adalimumab-Atto 100 Mg/Ml Prefilled Syringe [Amjevita]	2663904	2025
Adalimumab	0.4 Ml Adalimumab-Atto 100 Mg/Ml Prefilled Syringe [Amjevita]	2663907	2025
Adalimumab	0.4 Ml Adalimumab-Atto 100 Mg/Ml Auto-Injector [Amjevita]	2663910	2025
Adalimumab	0.8 Ml Adalimumab-Atto 100 Mg/Ml Auto-Injector [Amjevita]	2663915	2025
Adalimumab	0.8 Ml Adalimumab-Adaz 50 Mg/Ml Auto-Injector [Hyrimoz]	2665422	2025
Adalimumab	0.8 Ml Adalimumab-Adaz 50 Mg/Ml Prefilled Syringe [Hyrimoz]	2665426	2025
Adalimumab	0.8 Ml Adalimumab-Afzb 50 Mg/Ml Auto-Injector [Abrilada]	2668071	2025
Adalimumab	0.8 Ml Adalimumab-Afzb 50 Mg/Ml Prefilled Syringe [Abrilada]	2668078	2025

Adalimumab	0.4 Ml Adalimumab-Afzb 50 Mg/Ml Prefilled Syringe [Abrilada]	2668096	2025
Adalimumab	0.2 Ml Adalimumab-Aaty 100 Mg/Ml Prefilled Syringe	2668483	2025
Adalimumab	0.2 Ml Adalimumab-Aaty 100 Mg/Ml Prefilled Syringe [Yuflyma]	2668484	2025
Adalimumab	0.8 Ml Adalimumab-Aaty 100 Mg/Ml Auto-Injector	2668487	2025
Adalimumab	0.8 Ml Adalimumab-Aaty 100 Mg/Ml Auto-Injector [Yuflyma]	2668488	2025
Adalimumab	{3 (0.8 Ml Adalimumab-Aaty 100 Mg/Ml Auto-Injector [Yuflyma]) } Pack [Yuflyma	2668490	2025
Adalimumab	0.4 Ml Adalimumab-Ryvk 100 Mg/Ml Auto-Injector	2677780	2025
Adalimumab	0.4 Ml Adalimumab-Ryvk 100 Mg/Ml Auto-Injector [Simlandi]	2677787	2025
Adalimumab	0.4 Ml Adalimumab-Adbm 100 Mg/Ml Prefilled Syringe	2680743	2025
Adalimumab	0.4 Ml Adalimumab-Adbm 100 Mg/Ml Prefilled Syringe [Cyltezo]	2680746	2025
Adalimumab	0.4 Ml Adalimumab-Adbm 100 Mg/Ml Auto-Injector	2680748	2025
Adalimumab	0.4 Ml Adalimumab-Adbm 100 Mg/Ml Auto-Injector [Cyltezo]	2680750	2025
Adalimumab	{4 (0.4 Ml Adalimumab-Adbm 100 Mg/Ml Auto-Injector) } Pack	2680756	2025
Adalimumab	{4 (0.4 Ml Adalimumab-Adbm 100 Mg/Ml Auto-Injector [Cyltezo]) } Pack [Cyltezo	2680757	2025
Adalimumab	{6 (0.4 Ml Adalimumab-Adbm 100 Mg/Ml Auto-Injector) } Pack	2680759	2025
Adalimumab	{6 (0.4 Ml Adalimumab-Adbm 100 Mg/Ml Auto-Injector [Cyltezo]) } Pack [Cyltezo	2680760	2025
Adalimumab	0.4 Ml Adalimumab-Ryvk 100 Mg/Ml Prefilled Syringe	2688826	2025
Anakinra	0.67 Ml Anakinra 149 Mg/Ml Prefilled Syringe [Kineret]	727714	2022
Anakinra	0.67 Ml Anakinra 149 Mg/Ml Prefilled Syringe [Kineret]	727714	2023
Anakinra	0.67 Ml Anakinra 149 Mg/Ml Prefilled Syringe [Kineret]	727714	2024
Anakinra	0.67 Ml Anakinra 149 Mg/Ml Prefilled Syringe [Kineret]	727714	2025
Baricitinib	Baricitinib 2 Mg Oral Tablet [Olumiant]	2047247	2022
Baricitinib	Baricitinib 1 Mg Oral Tablet [Olumiant]	2205474	2022
Baricitinib	Baricitinib 2 Mg Oral Tablet [Olumiant]	2047247	2023
Baricitinib	Baricitinib 1 Mg Oral Tablet [Olumiant]	2205474	2023
Baricitinib	Baricitinib 2 Mg Oral Tablet [Olumiant]	2047247	2024
Baricitinib	Baricitinib 1 Mg Oral Tablet [Olumiant]	2205474	2024
Baricitinib	Baricitinib 4 Mg Oral Tablet [Olumiant]	2601725	2024
Baricitinib	Baricitinib 2 Mg Oral Tablet [Olumiant]	2047247	2025
Baricitinib	Baricitinib 1 Mg Oral Tablet [Olumiant]	2205474	2025
Baricitinib	Baricitinib 4 Mg Oral Tablet [Olumiant]	2601725	2025
Certolizumab	Certolizumab Pegol 200 Mg Injection [Cimzia]	795085	2022
Certolizumab	1 Ml Certolizumab Pegol 200 Mg/Ml Prefilled Syringe [Cimzia]	849599	2022
Certolizumab	Certolizumab Pegol 200 Mg Injection [Cimzia]	795085	2023
Certolizumab	1 Ml Certolizumab Pegol 200 Mg/Ml Prefilled Syringe [Cimzia]	849599	2023
Certolizumab	Certolizumab Pegol 200 Mg Injection [Cimzia]	795085	2024
Certolizumab	1 Ml Certolizumab Pegol 200 Mg/Ml Prefilled Syringe [Cimzia]	849599	2024
Certolizumab	Certolizumab Pegol 200 Mg Injection [Cimzia]	795085	2025
Certolizumab	1 Ml Certolizumab Pegol 200 Mg/Ml Prefilled Syringe [Cimzia]	849599	2025
Etanercept	Etanercept 25 Mg/Ml Injectable Solution [Enbrel]	261105	2022

Etanercept	1 Ml Etanercept 50 Mg/Ml Prefilled Syringe [Enbrel]	802652	2022
Etanercept	0.5 Ml Etanercept 50 Mg/Ml Prefilled Syringe [Enbrel]	809159	2022
Etanercept	1 Ml Etanercept 50 Mg/Ml Auto-Injector [Enbrel]	1653225	2022
Etanercept	1 Ml Etanercept 50 Mg/Ml Cartridge [Enbrel]	2182340	2022
Etanercept	0.5 Ml Etanercept 50 Mg/Ml Injection [Enbrel]	2392142	2022
Etanercept	1 Ml Etanercept 50 Mg/Ml Prefilled Syringe [Enbrel]	802652	2023
Etanercept	0.5 Ml Etanercept 50 Mg/Ml Prefilled Syringe [Enbrel]	809159	2023
Etanercept	1 Ml Etanercept 50 Mg/Ml Auto-Injector [Enbrel]	1653225	2023
Etanercept	1 Ml Etanercept 50 Mg/Ml Cartridge [Enbrel]	2182340	2023
Etanercept	0.5 Ml Etanercept 50 Mg/Ml Injection [Enbrel]	2392142	2023
Etanercept	1 Ml Etanercept 50 Mg/Ml Prefilled Syringe [Enbrel]	802652	2024
Etanercept	0.5 Ml Etanercept 50 Mg/Ml Prefilled Syringe [Enbrel]	809159	2024
Etanercept	1 Ml Etanercept 50 Mg/Ml Auto-Injector [Enbrel]	1653225	2024
Etanercept	1 Ml Etanercept 50 Mg/Ml Cartridge [Enbrel]	2182340	2024
Etanercept	0.5 Ml Etanercept 50 Mg/Ml Injection [Enbrel]	2392142	2024
Etanercept	1 Ml Etanercept 50 Mg/Ml Prefilled Syringe [Enbrel]	802652	2025
Etanercept	0.5 Ml Etanercept 50 Mg/Ml Prefilled Syringe [Enbrel]	809159	2025
Etanercept	1 Ml Etanercept 50 Mg/Ml Auto-Injector [Enbrel]	1653225	2025
Etanercept	1 Ml Etanercept 50 Mg/Ml Cartridge [Enbrel]	2182340	2025
Etanercept	0.5 Ml Etanercept 50 Mg/Ml Injection [Enbrel]	2392142	2025
Golimumab	0.5 Ml Golimumab 100 Mg/Ml Prefilled Syringe [Simponi]	848164	2022
Golimumab	1 Ml Golimumab 100 Mg/Ml Prefilled Syringe [Simponi]	1482814	2022
Golimumab	0.5 Ml Golimumab 100 Mg/Ml Auto-Injector [Simponi]	1653144	2022
Golimumab	1 Ml Golimumab 100 Mg/Ml Auto-Injector [Simponi]	1653166	2022
Golimumab	0.5 Ml Golimumab 100 Mg/Ml Prefilled Syringe [Simponi]	848164	2023
Golimumab	1 Ml Golimumab 100 Mg/Ml Prefilled Syringe [Simponi]	1482814	2023
Golimumab	0.5 Ml Golimumab 100 Mg/Ml Auto-Injector [Simponi]	1653144	2023
Golimumab	1 Ml Golimumab 100 Mg/Ml Auto-Injector [Simponi]	1653166	2023
Golimumab	0.5 Ml Golimumab 100 Mg/Ml Prefilled Syringe [Simponi]	848164	2024
Golimumab	1 Ml Golimumab 100 Mg/Ml Prefilled Syringe [Simponi]	1482814	2024
Golimumab	0.5 Ml Golimumab 100 Mg/Ml Auto-Injector [Simponi]	1653144	2024
Golimumab	1 Ml Golimumab 100 Mg/Ml Auto-Injector [Simponi]	1653166	2024
Golimumab	0.5 Ml Golimumab 100 Mg/Ml Prefilled Syringe [Simponi]	848164	2025
Golimumab	1 Ml Golimumab 100 Mg/Ml Prefilled Syringe [Simponi]	1482814	2025
Golimumab	0.5 Ml Golimumab 100 Mg/Ml Auto-Injector [Simponi]	1653144	2025
Golimumab	1 Ml Golimumab 100 Mg/Ml Auto-Injector [Simponi]	1653166	2025
Rilonacept	Rilonacept 220 Mg Injection [Arcalyst]	763457	2022
Rilonacept	Rilonacept 220 Mg Injection [Arcalyst]	763457	2023
Rilonacept	Rilonacept 220 Mg Injection [Arcalyst]	763457	2024
Rilonacept	Rilonacept 220 Mg Injection [Arcalyst]	763457	2025

Sarilumab	1.14 Ml Sarilumab 132 Mg/Ml Prefilled Syringe [Kevzara]	1923338	2022
Sarilumab	1.14 Ml Sarilumab 175 Mg/Ml Prefilled Syringe [Kevzara]	1923347	2022
Sarilumab	1.14 Ml Sarilumab 132 Mg/Ml Auto-Injector [Kevzara]	2003757	2022
Sarilumab	1.14 Ml Sarilumab 175 Mg/Ml Auto-Injector [Kevzara]	2003767	2022
Sarilumab	1.14 Ml Sarilumab 132 Mg/Ml Prefilled Syringe [Kevzara]	1923338	2023
Sarilumab	1.14 Ml Sarilumab 175 Mg/Ml Prefilled Syringe [Kevzara]	1923347	2023
Sarilumab	1.14 Ml Sarilumab 132 Mg/Ml Auto-Injector [Kevzara]	2003757	2023
Sarilumab	1.14 Ml Sarilumab 175 Mg/Ml Auto-Injector [Kevzara]	2003767	2023
Sarilumab	1.14 Ml Sarilumab 132 Mg/Ml Prefilled Syringe [Kevzara]	1923338	2024
Sarilumab	1.14 Ml Sarilumab 175 Mg/Ml Prefilled Syringe [Kevzara]	1923347	2024
Sarilumab	1.14 Ml Sarilumab 132 Mg/Ml Auto-Injector [Kevzara]	2003757	2024
Sarilumab	1.14 Ml Sarilumab 175 Mg/Ml Auto-Injector [Kevzara]	2003767	2024
Sarilumab	1.14 Ml Sarilumab 132 Mg/Ml Prefilled Syringe [Kevzara]	1923338	2025
Sarilumab	1.14 Ml Sarilumab 175 Mg/Ml Prefilled Syringe [Kevzara]	1923347	2025
Sarilumab	1.14 Ml Sarilumab 132 Mg/Ml Auto-Injector [Kevzara]	2003757	2025
Sarilumab	1.14 Ml Sarilumab 175 Mg/Ml Auto-Injector [Kevzara]	2003767	2025
Tocilizumab	0.9 Ml Tocilizumab 180 Mg/Ml Prefilled Syringe [Actemra]	1441530	2022
Tocilizumab	0.9 Ml Tocilizumab 180 Mg/Ml Auto-Injector [Actemra]	2106075	2022
Tocilizumab	0.9 Ml Tocilizumab 180 Mg/Ml Prefilled Syringe [Actemra]	1441530	2023
Tocilizumab	0.9 Ml Tocilizumab 180 Mg/Ml Auto-Injector [Actemra]	2106075	2023
Tocilizumab	0.9 Ml Tocilizumab 180 Mg/Ml Prefilled Syringe [Actemra]	1441530	2024
Tocilizumab	0.9 Ml Tocilizumab 180 Mg/Ml Auto-Injector [Actemra]	2106075	2024
Tocilizumab	0.9 Ml Tocilizumab 180 Mg/Ml Prefilled Syringe [Actemra]	1441530	2025
Tocilizumab	0.9 Ml Tocilizumab 180 Mg/Ml Auto-Injector [Actemra]	2106075	2025
Tocilizumab	0.9 Ml Tocilizumab-Aazg 180 Mg/Ml Auto-Injector [Tyenne]	2686050	2025
Tocilizumab	0.9 Ml Tocilizumab-Aazg 180 Mg/Ml Prefilled Syringe [Tyenne]	2686057	2025
Tofacitinib	Tofacitinib 5 Mg Oral Tablet [Xeljanz]	1357547	2022
Tofacitinib	24 Hr Tofacitinib 11 Mg Extended Release Oral Tablet [Xeljanz]	1741049	2022
Tofacitinib	Tofacitinib 10 Mg Oral Tablet [Xeljanz]	2048568	2022
Tofacitinib	24 Hr Tofacitinib 22 Mg Extended Release Oral Tablet [Xeljanz]	2273115	2022
Tofacitinib	Tofacitinib 1 Mg/Ml Oral Solution [Xeljanz]	2478437	2022
Tofacitinib	Tofacitinib 5 Mg Oral Tablet [Xeljanz]	1357547	2023
Tofacitinib	24 Hr Tofacitinib 11 Mg Extended Release Oral Tablet [Xeljanz]	1741049	2023
Tofacitinib	Tofacitinib 10 Mg Oral Tablet [Xeljanz]	2048568	2023
Tofacitinib	24 Hr Tofacitinib 22 Mg Extended Release Oral Tablet [Xeljanz]	2273115	2023
Tofacitinib	Tofacitinib 1 Mg/Ml Oral Solution [Xeljanz]	2478437	2023
Tofacitinib	Tofacitinib 5 Mg Oral Tablet [Xeljanz]	1357547	2024
Tofacitinib	24 Hr Tofacitinib 11 Mg Extended Release Oral Tablet [Xeljanz]	1741049	2024
Tofacitinib	Tofacitinib 10 Mg Oral Tablet [Xeljanz]	2048568	2024
Tofacitinib	24 Hr Tofacitinib 22 Mg Extended Release Oral Tablet [Xeljanz]	2273115	2024

Tofacitinib	Tofacitinib 1 Mg/Ml Oral Solution [Xeljanz]	2478437	2024
Tofacitinib	Tofacitinib 5 Mg Oral Tablet [Xeljanz]	1357547	2025
Tofacitinib	24 Hr Tofacitinib 11 Mg Extended Release Oral Tablet [Xeljanz]	1741049	2025
Tofacitinib	Tofacitinib 10 Mg Oral Tablet [Xeljanz]	2048568	2025
Tofacitinib	24 Hr Tofacitinib 22 Mg Extended Release Oral Tablet [Xeljanz]	2273115	2025
Tofacitinib	Tofacitinib 1 Mg/Ml Oral Solution [Xeljanz]	2478437	2025
Upadacitinib	24 Hr Upadacitinib 15 Mg Extended Release Oral Tablet [Rinvoq]	2196900	2022
Upadacitinib	24 Hr Upadacitinib 15 Mg Extended Release Oral Tablet [Rinvoq]	2196900	2023
Upadacitinib	24 Hr Upadacitinib 30 Mg Extended Release Oral Tablet [Rinvoq]	2590054	2023
Upadacitinib	24 Hr Upadacitinib 45 Mg Extended Release Oral Tablet [Rinvoq]	2596960	2023
Upadacitinib	24 Hr Upadacitinib 15 Mg Extended Release Oral Tablet [Rinvoq]	2196900	2024
Upadacitinib	24 Hr Upadacitinib 30 Mg Extended Release Oral Tablet [Rinvoq]	2590054	2024
Upadacitinib	24 Hr Upadacitinib 45 Mg Extended Release Oral Tablet [Rinvoq]	2596960	2024
Upadacitinib	24 Hr Upadacitinib 15 Mg Extended Release Oral Tablet [Rinvoq]	2196900	2025
Upadacitinib	24 Hr Upadacitinib 30 Mg Extended Release Oral Tablet [Rinvoq]	2590054	2025
Upadacitinib	24 Hr Upadacitinib 45 Mg Extended Release Oral Tablet [Rinvoq]	2596960	2025
Upadacitinib	Upadacitinib 1 Mg/Ml Oral Solution [Rinvoq]	2681019	2025

Note: This table contains a list of DMARDs included in the analysis and the RXCUI codes that identify each DMARD, obtained from the CMS Formulary Reference Files from 2022 to 2025.

eTable 2 Sensitivity to limiting the sample to plans operating throughout the study period

**Coverage generosity measures**[a]	**Stand-alone plans** Mean (95% CI)	**MA plans** Mean (95% CI)
Covering all mechanism of action, %[b]	12.6 (10, 15.1)	37.1 (33.3, 40.9)
Coverage share of TNF inhibitors (4), %[c,d]	48.2 (47.7, 48.6)	61.6 (60, 63.2)
Coverage share of T-cell costimulatory modulator (1), %[c,e]	18.9 (15.3, 22.5)	44.7 (40.8, 48.6)
Coverage share of IL-6 inhibitors (2), %[c,f]	32.6 (30.5, 34.7)	43.2 (40.9, 45.6)
Coverage share of JAK inhibitors (3), %[c,g]	51.9 (50.4, 53.4)	61.3 (59.5, 63.1)

Note: We show results after limiting the sample to plans operating throughout the study year (2022-2026), excluding 10,442 plan-year observations (44.5% of the original sample)

Abbreviations: TNF, Tumor necrosis factor; IL-6, Interleukin-6; JAK, Janus kinase; MA, Medicare Advantage; pp, percentage points; CI, confidence intervals

[a.] The table displays pooled yearly average of coverage generosity measure for stand-alone and Medicare Advantage plans, adjusting for secular trends (via year fixed effects) from 2022 to 2026. This estimate was clustered at the plan-level to account for within-plan clustering across the study period. We weighed each estimate by plan enrollment size, weighing plans with a larger number of beneficiaries more heavily.

[b] Proportion of plans covering at least one drug belong to each mechanism of action.

[c] Coverage share for each plan was determined by dividing the number of covered drugs in each mechanism of action (numerator) by the total number of drugs belonging to each class (denominator), which is indicated by the number in parentheses.

[d] TNF inhibitors included etanercept, adalimumab (or its biosimilar), golimumab, or certolizumab pegol

[e] Abatacept is the only T-cell costimulatory modulator included in the analysis

[f] IL-6 inhibitors included tocilizumab (or its biosimilar) and sarilumab

[g] JAK inhibitors included tofacitinib, baricitinib and upadacitinib

eTable 3 Sensitivity of main estimates to excluding non-representative plans

Coverage generosity measures[a]	Stand-alone plans<br>Mean (95% CI)	MA plans<br>Mean (95% CI)
Covering all mechanism of action, %[b]	10.4 (8.4, 12.5)	37 (33.9, 40.1)
Coverage share of TNF inhibitors (4), %[c,d]	48.5 (48.2, 48.9)	61.5 (60.1, 62.8)
Coverage share of T-cell costimulatory modulator (1), %[c,e]	19.4 (16.4, 22.3)	45 (41.8, 48.2)
Coverage share of IL-6 inhibitors (2), %[c,f]	31.3 (29.5, 33.1)	43.3 (41.3, 45.3)
Coverage share of JAK inhibitors (3), %[c,g]	53.4 (52.1, 54.6)	60.8 (59.2, 62.4)

Note: This table excludes non-representative plans (i.e., Special Needs Plans and regional Medicare Advantage plans); N = 5,461 (23% of all plan-year observations).

Abbreviations: TNF, Tumor necrosis factor; IL-6, Interleukin-6; JAK, Janus kinase; MA, Medicare Advantage; pp, percentage points; CI, confidence intervals

[a] The table displays pooled yearly average of coverage generosity measure for stand-alone and Medicare Advantage plans, adjusting for secular trends (via year fixed effects) from 2022 to 2026. This estimate was clustered at the plan-level to account for within-plan clustering across the study period. We weighed each estimate by plan enrollment size, weighing plans with a larger number of beneficiaries more heavily.

[b] Proportion of plans covering at least one drug belong to each mechanism of action.

[c] Coverage share for each plan was determined by dividing the number of covered drugs in each mechanism of action (numerator) by the total number of drugs belonging to each class (denominator), which is indicated by the number in parentheses.

[d] TNF inhibitors included etanercept, adalimumab (or its biosimilar), golimumab, or certolizumab pegol

[e] Abatacept is the only T-cell costimulatory modulator included in the analysis

[f] IL-6 inhibitors included tocilizumab (or its biosimilar) and sarilumab

[g] JAK inhibitors included tofacitinib, baricitinib and upadacitinib

eTable 4 Sensitivity of main estimates to excluding biosimilars

Coverage generosity measures[a]	Stand-alone plans	MA plans
	Mean (95% CI)	Mean (95% CI)
Covering all mechanism of action, %[b]	8.2 (6.6, 9.7)	28.8 (26.6, 31)
Coverage share of TNF inhibitors (4), %[c,d]	41.8 (40.9, 42.7)	58.7 (57.6, 59.8)
Coverage share of T-cell costimulatory modulator (1), %[c,e]	19.4 (16.4, 22.3)	44.8 (42.1, 47.5)
Coverage share of IL-6 inhibitors (2), %[c,f]	19.3 (17.7, 20.8)	28.7 (27.1, 30.3)
Coverage share of JAK inhibitors (3), %[c,g]	53.4 (52.1, 54.6)	60.5 (59.3, 61.8)

Note: In calculating these measures, we only considered the originator product, not biosimilars (adalimumab and tocilizumab biosimilars).

Abbreviations: TNF, Tumor necrosis factor; IL-6, Interleukin-6; JAK, Janus kinase; MA, Medicare Advantage; pp, percentage points; CI, confidence intervals

[a.] The table displays pooled yearly average of coverage generosity measure for stand-alone and Medicare Advantage plans, adjusting for secular trends (via year fixed effects) from 2022 to 2026. This estimate was clustered at the plan-level to account for within-plan clustering across the study period. We weighed each estimate by plan enrollment size, weighing plans with a larger number of beneficiaries more heavily.

[b] Proportion of plans covering at least one drug belong to each mechanism of action.

[c] Coverage share for each plan was determined by dividing the number of covered drugs in each mechanism of action (numerator) by the total number of drugs belonging to each class (denominator), which is indicated by the number in parentheses.

[d] TNF inhibitors included etanercept, adalimumab (or its biosimilar), golimumab, or certolizumab pegol

[e] Abatacept is the only T-cell costimulatory modulator included in the analysis

[f] IL-6 inhibitors included tocilizumab (or its biosimilar) and sarilumab

[g] JAK inhibitors included tofacitinib, baricitinib and upadacitinib

eTable 5 Sensitivity of main estimates to including IL-1 inhibitors

Coverage generosity measures[a]	Stand-alone plans	MA plans
	Mean (95% CI)	Mean (95% CI)
Covering all mechanism of action, %[b]	10.4 (8.4, 12.5)	37.6 (34.9, 40.2)
Coverage share of TNF inhibitors (4), %[c,d]	48.5 (48.2, 48.9)	61.7 (60.5, 62.8)
Coverage share of T-cell costimulatory modulator (1), %[c,e]	19.4 (16.4, 22.3)	44.8 (42.1, 47.5)
Coverage share of IL-6 inhibitors (2), %[c,f]	31.3 (29.5, 33.1)	42.7 (41.2, 44.3)
Coverage share of JAK inhibitors (3), %[c,g]	53.4 (52.1, 54.6)	60.5 (59.3, 61.8)
Coverage share of IL-1 inhibitors (2), %[c,h]	58.3 (57.4, 59.1)	67.6 (66.5, 68.7)

Note: In this table, we additionally considered coverage of IL-1 inhibitors in calculating the global measure of covering at least one drug in each mechanism of action.

Abbreviations: TNF, Tumor necrosis factor; IL-6, Interleukin-6; JAK, Janus kinase; MA, Medicare Advantage; pp, percentage points; CI, confidence intervals

[a.] The table displays pooled yearly average of coverage generosity measure for stand-alone and Medicare Advantage plans, adjusting for secular trends (via year fixed effects) from 2022 to 2026. This estimate was clustered at the plan-level to account for within-plan clustering across the study period. We weighed each estimate by plan enrollment size, weighing plans with a larger number of beneficiaries more heavily.

[b] Proportion of plans covering at least one drug belong to each mechanism of action.

[c] Coverage share for each plan was determined by dividing the number of covered drugs in each mechanism of action (numerator) by the total number of drugs belonging to each class (denominator), which is indicated by the number in parentheses.

[d] TNF inhibitors included etanercept, adalimumab (or its biosimilar), golimumab, or certolizumab pegol

[e] Abatacept is the only T-cell costimulatory modulator included in the analysis

[f] IL-6 inhibitors included tocilizumab (or its biosimilar) and sarilumab

[g] JAK inhibitors included tofacitinib, baricitinib and upadacitinib

[h] JAK inhibitors included anakinra and rilonacept

eTable 6 Other restrictions on covered drugs by plan type

	Any utilization management[a]		Placement on specialty tier[b]	
	**Stand-alone**	**MA-PD**	**Stand-alone**	**MA-PD**
Abatacept	100.0	89.1	100.0	96.6
Adalimumab	100.0	94.9	100.0	91.4
Anakinra	100.0	86.7	100.0	97.3
Baricitinib	100.0	57.1	100.0	94.2
Certolizumab	100.0	100.0	100.0	96.3
Etanercept	100.0	95.8	100.0	95.5
Golimumab	100.0	83.7	100.0	98.1
Rilonacept	100.0	93.5	100.0	95.5
Sarilumab	100.0	85.8	100.0	95.1
Tocilizumab	100.0	91.6	100.0	96.2
Tofacitinib	100.0	98.7	100.0	95.2
Upacitinib	100.0	95.1	100.0	95.7

Abbreviations: MA-PD, Medicare Advantage Part D plans

Note: This table displays the proportion of plans applying additional utilization restrictions for drug, conditional on coverage.

[a] Any prior authorization, step therapy, or quantity limits applied for the drug

[b] Placement on tier 5 or 6 of the formulary

eTable 7 Stratified analysis by Census region

West

Coverage generosity measures[a]	Stand-alone Mean (95% CI)	MA plans Mean (95% CI)
Covering all mechanism of action, %[b]	10.1 (5, 15.3)	51.6 (45.4, 57.8)
Coverage share of TNF inhibitors (4), %[c,d]	48.4 (47.4, 49.4)	67.4 (64.5, 70.2)
Coverage share of T-cell costimulatory modulator (1), %[c,e]	21.3 (12.5, 30.2)	60.7 (55.3, 66.2)
Coverage share of IL-6 inhibitors (2), %[c,f]	31 (26.4, 35.5)	48.3 (43.4, 53.2)
Coverage share of JAK inhibitors (3), %[c,g]	51.6 (48.1, 55.1)	68.5 (64.9, 72.1)

Midwest

Coverage generosity measures[a]	Stand-alone Mean (95% CI)	MA plans Mean (95% CI)
Covering all mechanism of action, %[b]	10.3 (7.1, 13.5)	36.4 (31.1, 41.7)
Coverage share of TNF inhibitors (4), %[c,d]	48.6 (48, 49.2)	59.6 (57.5, 61.7)
Coverage share of T-cell costimulatory modulator (1), %[c,e]	17.3 (13.4, 21.2)	42.3 (36.4, 48.2)
Coverage share of IL-6 inhibitors (2), %[c,f]	30.6 (27.1, 34)	41.8 (38.4, 45.1)
Coverage share of JAK inhibitors (3), %[c,g]	53.5 (51.4, 55.7)	58.4 (56.3, 60.4)

South

Coverage generosity measures[a]	Stand-alone Mean (95% CI)	MA plans Mean (95% CI)
Covering all mechanism of action, %[b]	10.4 (6.7, 14)	30.9 (27.6, 34.1)
Coverage share of TNF inhibitors (4), %[c,d]	48.5 (48, 49)	58.9 (57.4, 60.3)
Coverage share of T-cell costimulatory modulator (1), %[c,e]	18.2 (13.7, 22.8)	36.9 (33.3, 40.4)
Coverage share of IL-6 inhibitors (2), %[c,f]	31.2 (28.3, 34.1)	39.7 (38.4, 41)
Coverage share of JAK inhibitors (3), %[c,g]	53.5 (51.7, 55.3)	54.7 (53.4, 56)

Northeast

Coverage generosity measures[a]	Stand-alone Mean (95% CI)	MA plans Mean (95% CI)
Covering all mechanism of action, %[b]	11.2 (6.1, 16.2)	40.4 (33.4, 47.3)
Coverage share of TNF inhibitors (4), %[c,d]	48.7 (47.6, 49.8)	65.6 (62.7, 68.6)
Coverage share of T-cell costimulatory modulator (1), %[c,e]	22.4 (14.9, 29.8)	45.4 (38.1, 52.6)
Coverage share of IL-6 inhibitors (2), %[c,f]	32.8 (29, 36.6)	49.9 (46.6, 53.2)
Coverage share of JAK inhibitors (3), %[c,g]	54.8 (52.4, 57.2)	67.6 (65.7, 69.5)

Note: In these tables, we show results separately for plans operating in each Census region. For local plans, we were able to delineate exact regions of operation by state; for regional plans that serve across regions, they may be included in the denominator across regions.
Abbreviations: TNF, Tumor necrosis factor; IL-6, Interleukin-6; JAK, Janus kinase; MA, Medicare Advantage; pp, percentage points; CI, confidence intervals

[a.] The table displays pooled yearly average of coverage generosity measure for stand-alone and Medicare Advantage plans, adjusting for secular trends (via year fixed effects) from 2022 to 2026. This estimate was clustered at the plan-level to account for within-plan clustering across the study period. We weighed each estimate by plan enrollment size, weighing plans with a larger number of beneficiaries more heavily.

[b] Proportion of plans covering at least one drug belong to each mechanism of action.

[c] Coverage share for each plan was determined by dividing the number of covered drugs in each mechanism of action (numerator) by the total number of drugs belonging to each class (denominator), which is indicated by the number in parentheses.

[d] TNF inhibitors included etanercept, adalimumab (or its biosimilar), golimumab, or certolizumab pegol

[e] Abatacept is the only T-cell costimulatory modulator included in the analysis

[f] IL-6 inhibitors included tocilizumab (or its biosimilar) and sarilumab

[g] JAK inhibitors included tofacitinib, baricitinib and upadacitinib

[h] JAK inhibitors included anakinra and rilonacept

eTable 8 Using "at least 2 drugs" threshold to evaluate within-class generosity

	**Stand-alone plans**	**MA plans**
**Coverage generosity measure**	Mean (95% CI)	Mean (95% CI)
Covering at least 2 TNF Inhibitors, %[a]	93.9 (92.5, 95.4)	96.1 (95.6, 96.6)
Covering one T-cell Costimulation Modulator, %[b]	19.4 (16.4, 22.3)	44.8 (42.1, 47.5)
Covering at least 2 IL-6 Inhibitors, %[c]	0.1 (0, 0.2)	9 (7.1, 11)
Covering at least 2 JAK Inhibitors, %[d]	60 (56.4, 63.6)	72.5 (70.2, 74.8)

Abbreviations: TNF, Tumor necrosis factor,; IL-6, Interleukin-6; JAK, Janus kinase; MA, Medicare Advantage; pp, percentage points

[a] Proportion of plans covering at least two of the following: Etanercept, Adalimumab, Golimumab, or Certolizumab Pegol

[b] Proportion of plans covering Abatacept, which is the sole drug included in this class.

[c] Proportion of plans covering both Tocilizumab and Sarilumab

[d] Proportion of plans covering at least two of the following: Tofacitinib, Baricitinib, Upadacitinib
